# Community Oriented Primary Care

**DOI:** 10.4102/phcfm.v8i1.1202

**Published:** 2016-09-15

**Authors:** Srini Govender

**Affiliations:** 1 Family Medicine and Primary Care, Stellenbosch University, South Africa

This review looks at two closely related books by the same author on community-oriented primary care (COPC).

The first book on community-oriented primary care by Tessa Marcus is a simple, practical and well-structured introduction to a complex topic that has long been in the shadow of bio-medical medicine. It provides an overview of the concepts and principles of COPC together with the integration of how and why individuals, families and societies are important.

It refers to primary care as ‘first line, basic health care and COPC as primary care where professionals from different disciplines and approaches work together with organizations and people in defined communities to identify and respond systematically to health and health related needs in order to improve health’.

‘COPC is an active partnership between health care providers and health care users. It is designed to begin with individuals and families in their homes and their communities, so that they can create opportunities to improve health, contain illness and disease, and change the system of health care’.

The book is well structured and actively involves the reader with well-designed, interactive and simple exercises. There is also the opportunity to access more web-based detail from the publisher by the use of QR codes.

It has three main topics.

Topic 1 is about the concept of COPC and begins with an outline of five principles:

- Local and health institutional analysis.
- Comprehensive care, which also alludes to the social determinants of health.
- Equity: providing accessible, appropriate, affordable and relevant health care.
- Practice with science
- Service integration around users

Topic 2 deals with the concept of community as individuals and families in time, space and place. It expands on our understanding of the role of the individual and families in society and the complex interaction over time, place and space. While this section is rich, important and detailed, it may seem somewhat abstract and theoretical for those wanting a more pragmatic approach to COPC.

Topic 3 elaborates on the idea of social stratification within the community. It explains how the concepts of age, gender, disability, racial and social stratification affect the implementation of each of the principles of COPC.

Overall, this book is an important theoretical overview of the concept of COPC and its importance in health care provision. It provides a detailed theoretical map with practical examples of why individuals, families and societies, and their complex relationships, are inextricably linked to health and well-being.

The second book is an excellent introduction to the development of COPC. It is essential reading for those interested in COPC whether to get a theoretical grasp or when implementing an intervention. Rather than a practical ‘How to Implement COPC’, it sets the tone and background context. It is an overview of the social, economic and political history of the development of COPC in South Africa and its wider global adoption. It is easy to read and well laid out with four sections.

Section 1 is about the background and context of COPC. COPC is an approach to health care that developed in response to a confluence of social, economic and political pressure. Sidney and Emily Kark were the two pioneering South African doctors who made social context and relationships an integral part of the practice of the health service. They combined the principles of primary health care and public health by gathering social scientific and epidemiological data in defined geographical areas which informed health service provision.

Section 2 tells the story of Pholela and doing COPC for the first time in the 1930s. Little was known about the health needs of the population of about 30 000. They faced dire poverty made worse by the great depression and the migrant labour system. This was a story of vision and commitment, good leadership and teamwork.

Section 3 expands on how the COPC movement extended throughout South Africa. Pholela was the start of what became known as the Health Centre Movement, which spread across South Africa and to other parts of the world. This was possible because of a brief period of government support following the Gluckman commission report. This report argued that it was wrong to put all government efforts into more hospital beds and that the focus needed to be on primary health care.

Section 4 is about the expansion of COPC to the rest of the world. With the new Apartheid government and the systematic dismantling of the local COPC initiative, the Karks together with other champions moved on to other parts of the world – Israel, England and the United States, where they developed and adapted training and implementation of COPC. Paradoxically, Apartheid was the trigger for the spread of COPC across the world. There is more detail on the implementation of COPC in the United States, Cuba, Spain and Brazil.

Overall, the book provides a clear and concise summary of the historical development of COPC across the globe. It is also an important reminder, now more than ever, that social determinants of health and primary care are crucial in facing our current health challenges.

